# Ultra-Sensitive Ultrasound

**DOI:** 10.7759/cureus.7751

**Published:** 2020-04-20

**Authors:** Maranda Q Herner, Anna Maw

**Affiliations:** 1 Internal Medicine, University of Colorado- Anschutz Medical Campus, Aurora, USA

**Keywords:** lung ultrasound, point-of-care-ultrasound, pulmonary edema, diagnostic accuracy

## Abstract

Lung ultrasound (LUS) is a dynamic, real-time, non-invasive bedside tool that offers increased sensitivity over standard imaging modalities in identifying pulmonary edema. This case highlights acute post-operative hypoxia secondary to pulmonary edema that was initially missed by chest radiography (CXR) and chest computed tomography (CT). The edema was diagnosed first on same day by bedside LUS, later seen on next day follow-up CXR and resolved with diuresis. LUS has demonstrated superior accuracy compared to CXR, but scant evidence compares it to CT. This case presentation serves to increase awareness of LUS as a highly sensitive and easy-to-use diagnostic tool for hospital providers in the evaluation of acute hypoxia.

## Introduction

A multicenter study recently identified pulmonary edema, atelectasis and pleural effusions as frequent causes of post-operative hypoxia requiring over 24-hours of supplemental oxygen [1]. Lung ultrasound (LUS) is known to be more accurate than other bedside tools in the detection of pulmonary edema including auscultation and chest radiography (CXR) [2]. Its accuracy compared to chest computed tomography (CT) has not been well studied. This report presents a case of acute hypoxia secondary to pulmonary edema found on bedside LUS that was initially missed by both CXR and chest CT.

## Case presentation

**Figure 1:**
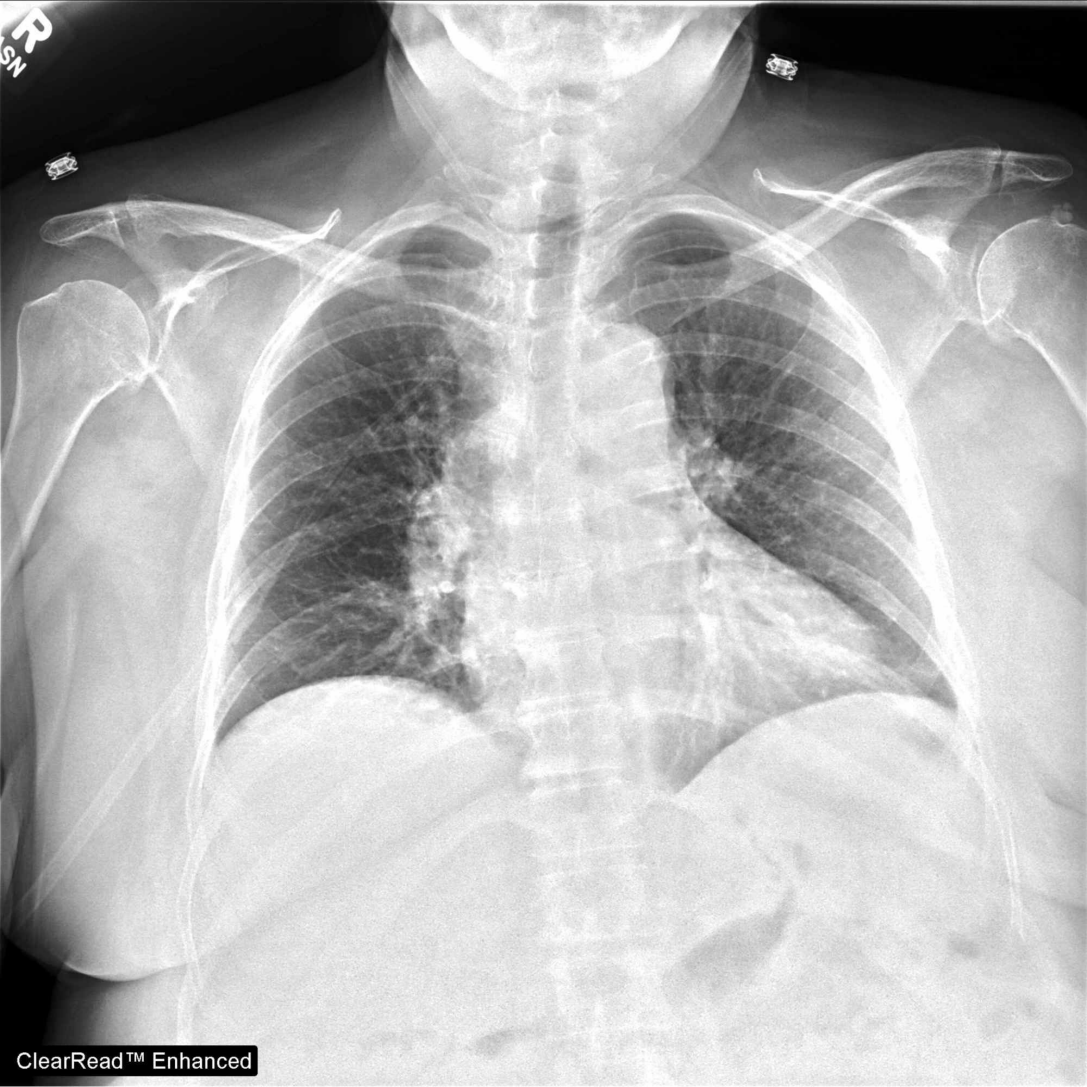
Initial Chest Radiography (CXR), with no evidence of pulmonary edema

A 66-year-old, non-obese woman with no significant past medical history initially presented with right knee pain. Synovial fluid analysis was consistent with septic arthritis for which she received joint wash out under general anesthesia. Postoperatively, she developed transient chest pain, tachypnea and new hypoxia with PaO2 of 59mmHg on room air, requiring 5L/min of oxygen to maintain a saturation above 90%. Her initial post-operative CXR was read as nonspecific with bibasilar and perihilar opacities concerning for atelectasis or aspiration (Figure 1).

The radiology report of the same day pulmonary embolism (PE) protocol chest CT was negative for PE, positive for bibasilar atelectasis and small bilateral effusions but without mention of pulmonary edema. Her troponin was negative, brain natriuretic peptide (BNP) was 253, and arterial blood gas showed no hypercarbia and normal A-a gradient. Electrocardiogram was non-ischemic with sinus tachycardia. Echocardiogram showed right ventricular systolic pressure elevation to 41mmHg but no evidence of left ventricular systolic or diastolic dysfunction. Even after her chest pain resolved and her respiratory rate normalized, she remained hypoxemic.

**Figure 2:**
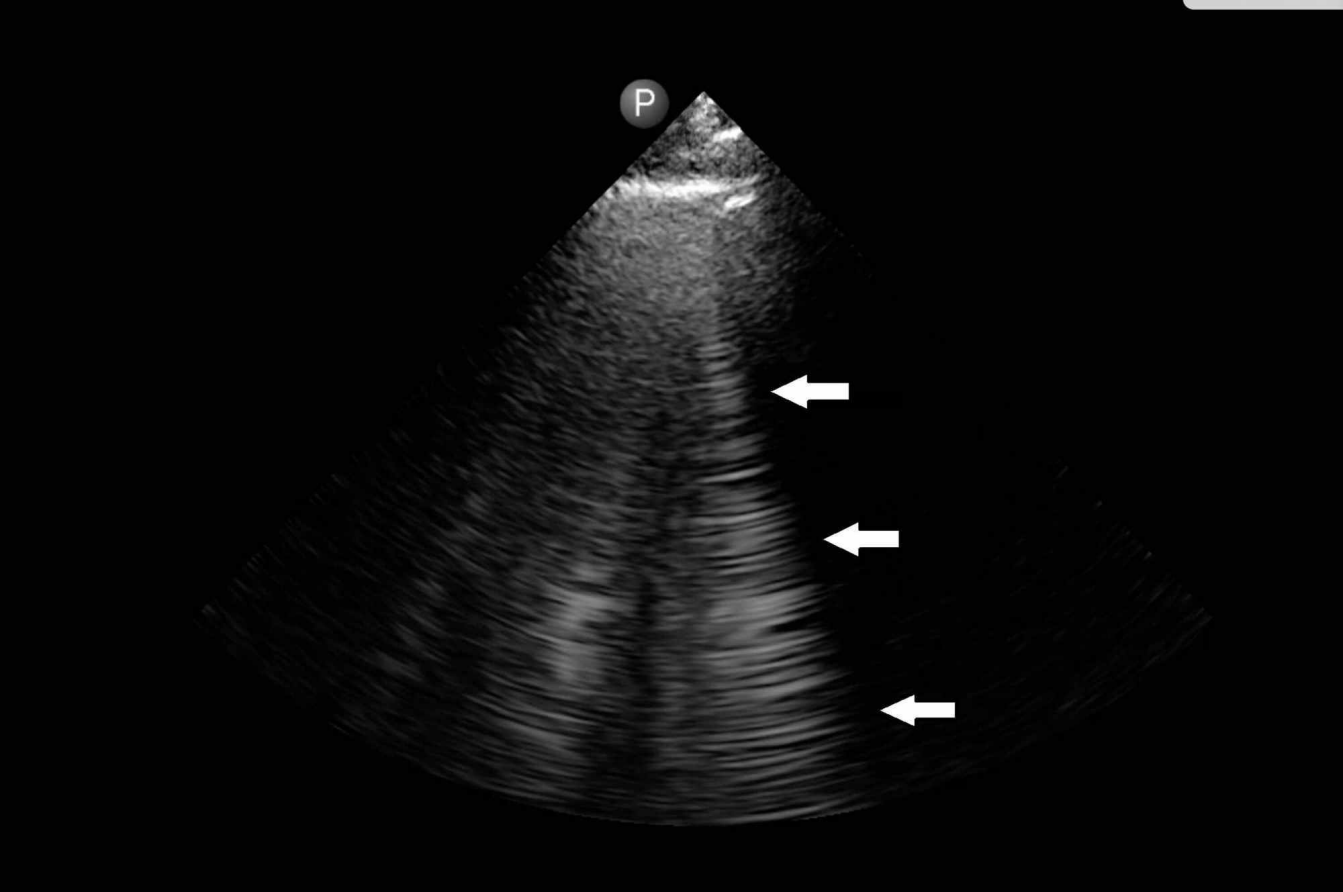
Lung Ultrasound, with multiple comet tails (also known as B-lines) One vertical comet tail (B-line) is indicated by the three white arrows.

Clinically, her 16-pound weight increase from admission, reported orthopnea, and mildly elevated brain natriuretic peptide (BNP) prompted a same day LUS which demonstrated multiple bilateral comet tails (Figure 2).

**Figure 3:**
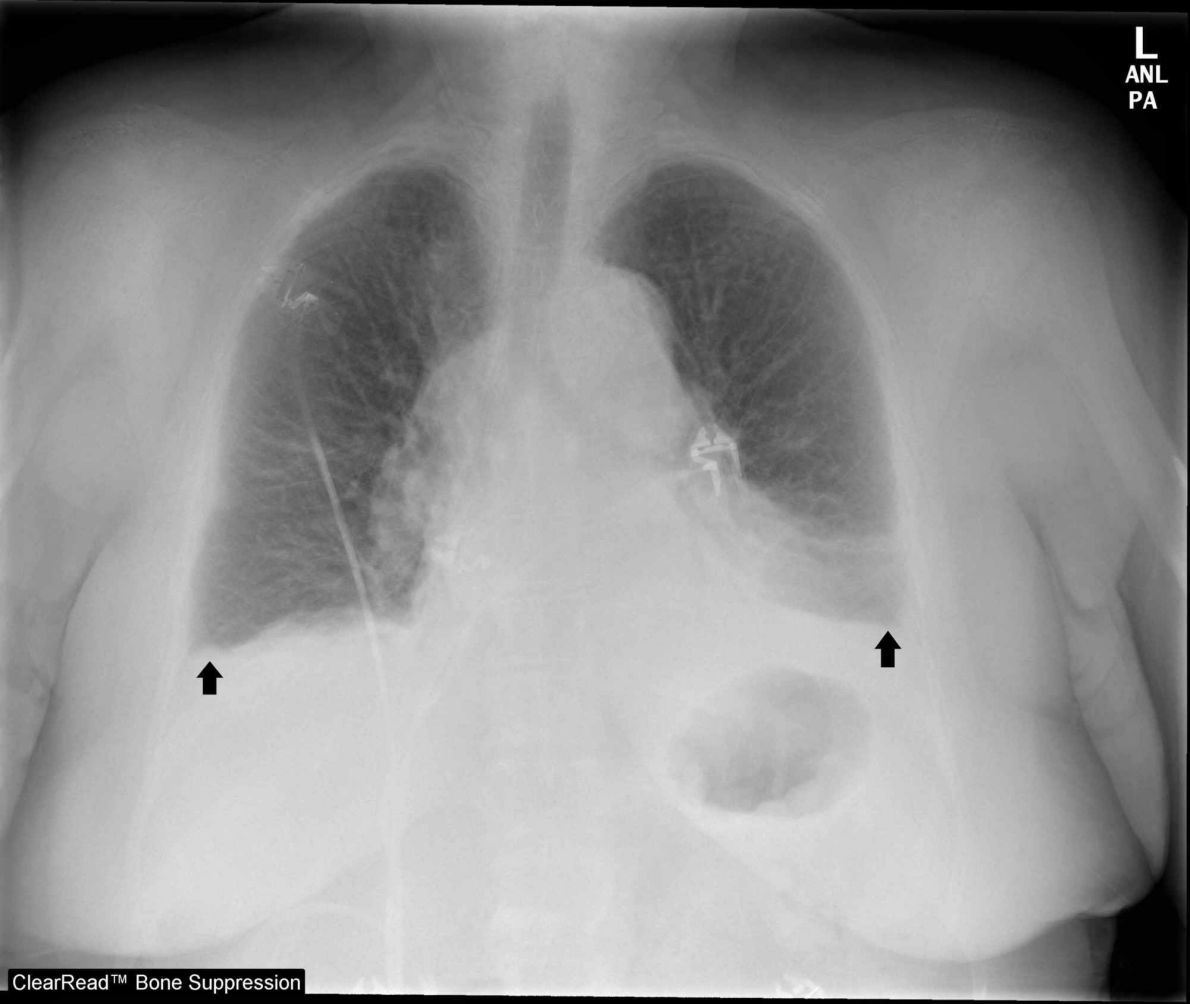
One Day Later, Repeat Chest Radiography (CXR) Black arrows identify bilateral pleural effusions.  CXR also notable for diffuse vascular congestion with pulmonary edema.

While her oxygen requirements remained elevated and unchanged the following day, a repeat CXR noted enlarging bilateral effusions and evidence of vascular congestion consistent with pulmonary edema (Figure 3).

The team suspected her hypoxemia resulted from a combination of pulmonary edema and hypoventilation related to atelectasis. Her mild pulmonary hypertension and mildly elevated BNP may reflect cardiogenic edema, though the echocardiogram was non-confirmatory for left ventricular dysfunction. Medication induced diuresis led to improvement on serial CXRs and resolution of her hypoxemia, consistent with pulmonary edema as the underlying etiology. At post-discharge follow up, she had returned to pre-admission weight and exhibited a 95% oxygen saturation on room air.

## Discussion

As a portable, dynamic and minimally invasive tool, LUS can identify pulmonary edema in acute hypoxic respiratory failure (AHRF) through vertically oriented, hyperechoic lines of artifact termed comet tails or B-lines, as seen in our patient (Figure 2). In an observational study, multiple B-lines on LUS demonstrated a sensitivity of 97% and specificity of 95% for pulmonary edema differentiated from other causes of AHRF [3].

CXR exhibits a false negative rate of 20% for detecting pulmonary edema and has been shown in a meta-analysis to be less sensitive than LUS [2]. Further, LUS provides a more accurate, real-time lung assessment as CXR has a known lag time in revealing both the initial presence and the resolution of pulmonary edema [4]. The dynamic nature of LUS has been well demonstrated in studies of patients receiving hemodialysis. LUS performed immediately before and after hemodialysis showed significant reduction in B-lines post dialysis, which correlated with weight reductions [5]. Studies monitoring response to diuretics have also noted LUS to demonstrate evidence of improvement sooner than CXR, highlighting LUS’s earlier detection of changes in the dynamic process of pulmonary edema [6]. Consistent with these observations, this patient’s first CXR failed to demonstrate the vascular congestion seen on the next day CXR. LUS offered this patient a more sensitive diagnostic tool for her early pulmonary edema.

While prior studies evaluating the accuracy of LUS have used chest CT as the reference standard, this case uniquely demonstrates failure of CT to diagnose acute pulmonary edema [2]. This raises the question of the sensitivity of LUS compared to CT which can be addressed in future studies.

## Conclusions

LUS is a highly accurate, practical and dynamic bedside tool that should be considered in the evaluation of any patient with AHRF, particularly in early presentations such as in the post-operative patient. In this case, initial CXR and CT chest failed to capture evidence of pulmonary edema. However, the multiple comet tails on LUS and the patient's brisk response to diuretic therapy indicate that pulmonary edema was in fact present. This case supports the more frequent use of LUS as a highly accurate and evidence-based diagnostic tool for AHRF. Diagnoses can be accelerated, and subsequent treatment interventions applied sooner when LUS, being a new, more sensitive tool for pulmonary edema, is utilized early. The incorporation of LUS in routine hospital practices can significantly improve diagnostic accuracy and therefore patient care.
